# Miss Blackwell, M. D.

**Published:** 1850-10

**Authors:** 


*Miss Blackwell, M. D.*—The movements of this estimable lady, and intrepid pioneer in the cause of female medical education, will continue to be a subject of interest with the Medical Profession. A private letter has been transmitted to us by a mutual friend, which we are not at liberty to insert in full, by which we learn that she has continued to prosecute her studies in Paris, up to July last. The disease of one of her eyes, contracted from a patient under her observation, has proved a serious calamity, the sight being nearly destroyed.

In July she was at *Grafenburg*, at the hydropathic establishment of *Priessnitz*, partly to try the effects of his system upon herself, and, partly, to study the effects of his system upon the numerous patients congregating there, with a view to ascertain what success is really attained, and to determine how much is to be attributed to the therapeutic action of water, and how much to the general hygienic conditions under which the patients are placed.

She states that she has received a courteous invitation to pass several months in London, every facility for attending the hospitals and schools having been promised; and that it is her intention to avail herself of this opportunity to institute a comparison between French and British practice.

